# Molecular characterization of extracellular vesicles derived from follicular fluid of women with and without PCOS: integrating analysis of differential miRNAs and proteins reveals vital molecules involving in PCOS

**DOI:** 10.1007/s10815-023-02724-z

**Published:** 2023-01-25

**Authors:** Yuqin Yang, Peng Lang, Xiaolan Zhang, Xun Wu, Shanren Cao, Chun Zhao, Rong Shen, Xiufeng Ling, Ye Yang, Junqiang Zhang

**Affiliations:** Department of Reproductive Medicine, Women’s Hospital of Nanjing Medical University, Nanjing Maternity and Child Health Care Hospital, Nanjing, China

**Keywords:** Polycystic ovary syndrome, Follicular fluid, Extracellular vesicles, MicroRNA, Proteomics, Bioinformatic

## Abstract

**Purpose:**

To elucidate the characterization of extracellular vesicles (EVs) in the follicular fluid-derived extracellular vesicles (FF-EVs) and discover critical molecules and signaling pathways associating with the etiology and pathobiology of PCOS, the differentially expressed miRNAs (DEmiRNAs) and differentially expressed proteins profiles (DEPs) were initially explored and combinedly analyzed.

**Methods:**

First, the miRNA and protein expression profiles of FF-EVs in PCOS patients and control patients were compared by RNA-sequencing and tandem mass tagging (TMT) proteomic methods. Subsequently, Gene Ontology and the Kyoto Encyclopedia of Genes and Genomes were used to analyze the biological function of target genes of DEmiRNAs and DEPs. Finally, to discover the functional miRNA-target gene-protein interaction pairs involved in PCOS, DEmiRs target gene datasets and DEPs datasets were used integratedly.

**Results:**

A total of 6 DEmiRNAs and 32 DEPs were identified in FF-EVs in patients with PCOS. Bioinformatics analysis revealed that DEmiRNAs target genes are mainly involved in thiamine metabolism, insulin secretion, GnRH, and Apelin signaling pathway, which are closely related to the occurrence of PCOS. DEPs also closely related to hormone metabolism processes such as steroid hormone biosynthesis. In the analysis integrating DEmiRNAs target genes and DEPs, two molecules, GRAMD1B and STPLC2, attracted our attention that are closely associated with cholesterol transport and ceramide biosynthesis, respectively.

**Conclusion:**

Dysregulated miRNAs and proteins in FF-EVs, mainly involving in hormone metabolism, insulin secretion, neurotransmitters regulation, adipokine expression, and secretion, may be closely related to PCOS. The effects of GRAMD1B and STPLC2 on PCOS deserve further study.

## Introduction

Polycystic ovary syndrome (PCOS) is regarded as the most common and complex endocrine disorder, affecting ∼6% to 20% of reproductive aged women [1, 2]. It generally manifests with ovulatory dysfunction, fertility decline, clinical and biochemical androgen excess, and polycystic ovaries [3–6].

Assisted reproductive technology (ART) has become an integral part of modern medicine that brings hope for PCOS infertility patients [7]. By using medications that work on super-ovulating to obtain more eggs and inducing multiple follicles to mature, PCOS infertility patients can get pregnant through clinical in vitro fertilization technology. Although ART has improved the pregnancy rate of PCOS patients, many studies have confirmed that PCOS patients have lower egg quality and eggs with lower pregnancy potential, which seriously affects the outcome of assisted reproductive technology [8]. Nevertheless, what is needed to be soberly aware is that the molecular details of PCOS remains unclear.

Follicular development involves a complex network of interacting cellular signals. Follicular fluid (FF) that is mainly formed by the secretion of granulosa cells, follicular membrane cells, and oocytes; and the diffusion of plasma components from capillaries to antrum [9, 10] provides an important microenvironment for oocyte development and maturation.

Exosomes, released from cells, are spherical or cup-shaped vesicles with a double membrane, with a diameter of approximately 40–100 nm. Studies have shown that exosomes are widely found in blood, human milk, placenta, and amniotic fluid [11, 12]. Exosomes contain a variety of regulatory molecules, such as nucleic acids, mRNA, microRNAs (miRNA), proteins, and lipids. These components of exosomes can transfer between different types of cells to regulate corresponding biological processes and signaling pathways, e.g., affecting immunity, intercellular communication, cell proliferation, cell differentiation, and metabolic diseases [13–15].

A few of studies have shown that exosomes exist in FF [16] and act as information transmitters in somatic cells and oocyte communication by transferring a variety of proteins, lipids, miRNAs, and circRNAs [17–20]. For instant, in 2012, da Silveira et al. described the exosomes containing miRNA and protein in horse FF for the first time and further discussed the role of miRNAs related to hormone regulation in different ovarian follicular cells [21]; later, some scholars successively isolated exosomal miRNA from bovine FF and further proved evidences that it can support cumulus expansion [17, 22]. Recently, a study found that the reduction of circLDLR in follicular fluid-derived exosomes (FF-EVs) derepresses the function of miR-1294 and inhibits estradiol production via CYP19A1 in PCOS [18]. Another study found that the exosomal miR-424-5p derived from PCOS FF inhibits granulosa cell proliferation and induces their senescence by targeting CDCA4-mediated Rb/E2F1 signaling [19]. Wang et al. found abnormal expression of long non-coding RNA (LncRNA) in the FF-EVs of PCOS patients [20]. A proteomics study on FF-EVs found that the S100-A9 protein in exosomes from FF promotes inflammation by activating the NF-κB pathway in PCOS [23]. Taken together, these reports reflect that FFEs and their components play an important regulatory role in the growth and development of oocytes.

It is well known that miRNAs subtly influence a vast number of proteins involved in most key biological processes via binding to the 3′-untranslated region (3′UTR) of target genes for cleavage or translational repression [24, 25]. Nevertheless, although, in theory, a single miRNA can indeed dampen levels of hundreds of proteins [26], the effects of miRNAs on proteins are usually quite modest, changing their expression levels by less than twofold.

Integrated analysis of miRNA and protein expression profiles can still be helpful to identify the functional miRNA-target gene-protein interaction pairs involved in regulating specific biological processes. Previously, although there were a few studies on expression profiles analysis of FF-EVs derived from PCOS patients such as, miRNA, lncRNA, or protein, there are very few studies on the above specimens by performing combined analysis of miRNA and protein expression profiles.

In this study, we performed combined analysis of the miRNAs and protein expression profiles of follicular fluid-derived extracellular vesicles (FF-EVs; we called exosomes and microvesicles as “extracellular vesicles” in this study because of the overlapping size range of different types of extracellular vesicles as well as lack of specific marker for distinguishing them) collected from PCOS patients and healthy women in order to accurately seize the key miRNAs that can cause the changes of protein levels in the FF-EVs of PCOS patients and to further discover molecular details led to PCOS. We found that 6 miRNAs and 32 proteins were significantly differentially expressed between PCOS and the control group. We noticed that the hormone-related metabolic and steroid hormone biosynthesis processes were enriched in both differentially expressed miRNA (DEmiRNAs) and differentially expressed proteins (DEPs) data sets. Especially, we noticed two critical molecules, GRAMD1B (GRAM Domain Containing 1B) and SPTLC2 (Serine Palmitoyltransferase Long Chain Base Subunit 2), that are closely associated with PCOS. Our study will help in amendment and improvement of understanding of FF-EVs in patients with PCOS, thus laying a foundation for future research on the role of miRNAs and proteins in the pathogenesis of PCOS, which may be used as potential biomarkers for the diagnosis and treatment of PCOS.

## Results

### Characterization and validation of FF-EVs

**Fig. 1:**
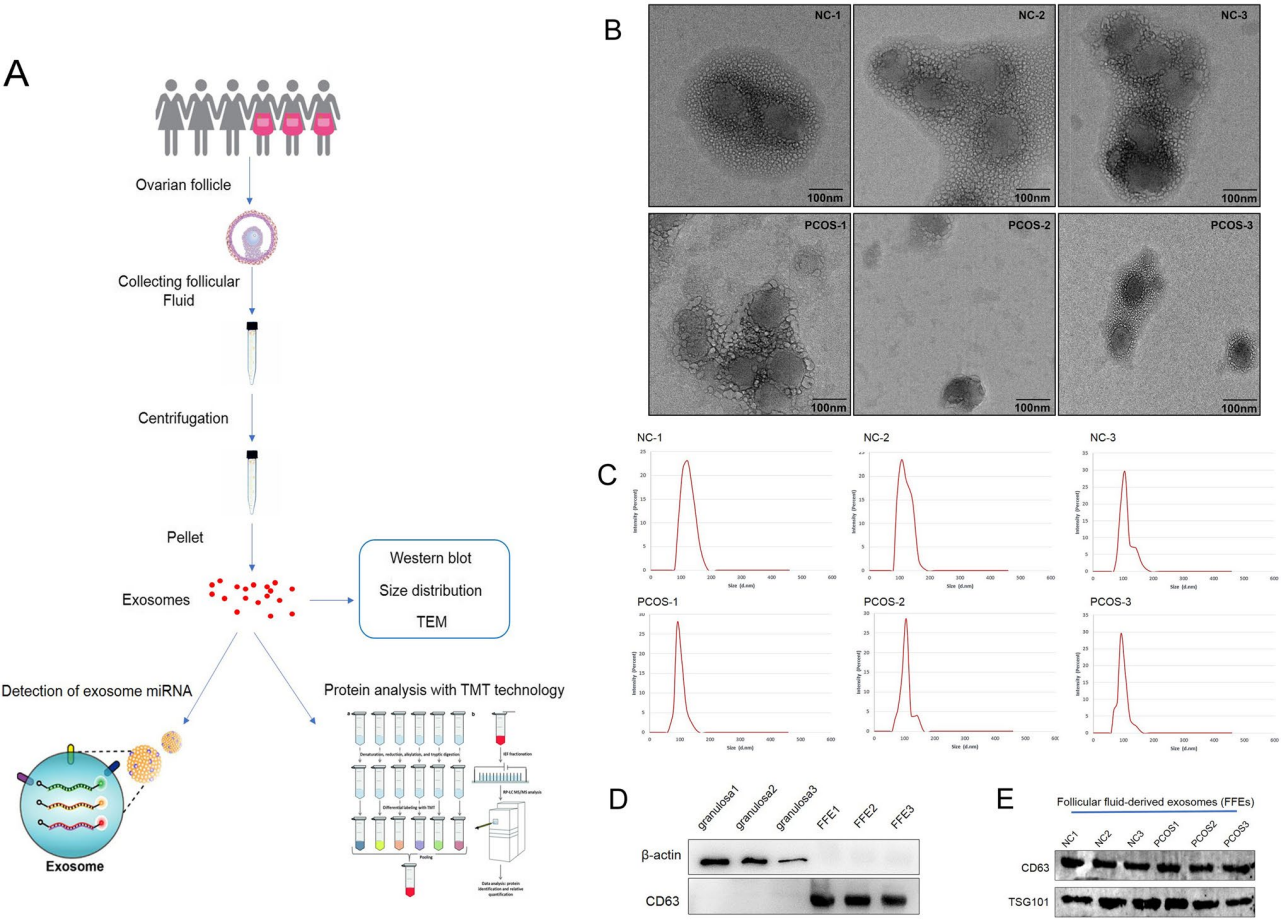
Isolation and identification of FF-EVs. (**A**) The route and methods of this research. (**B**) TEM results of extracellular vesicles derived from FF of PCOS patients and healthy women. (**C**) NTA results profile of extracellular vesicles from ovarian FF of PCOS patients and healthy women. (**D**) Western blotting is used to verify isolated FF-EVs without granulosa contamination. CD63 and β-actin were used as primary antibodies, respectively. (**E**) The extracellular vesicles isolated from ovarian FF of PCOS patients and healthy women were verified by western blot analysis

The clinical characteristics of PCOS patients and control women were listed in Table 1. Based on the results of statistical difference analysis, the levels of LH, E2, T, PRL, and AMH in PCOS group were statistically higher than that in control group. By contrast, the mean infertility duration was shorter in PCOS group than that in control group. The route and methods of this study were shown in Fig. 1A. To characterize FF-EVs, due to their nanosize, transmission electron microscope (TEM) was performed to show and assess their morphology. As shown in Fig. 1B, FF-EVs were round or oval vesicles. The density of FF-EVs was varied from individual to individual. The nanoparticle tracking analysis (NTA) results showed that the diameters of FF-EVs obtained from the 6 samples sized ranging from 30 to 150 nm (Fig. 1C), which was consistent with the characteristic sizes (30–120 nm) of extracellular vesicles. Furthermore, to validate the purity of the extracted FF-EVs, the exosomal markers, CD63 and TSG101, as well as non-exosomal markers, β-actin, were detected by western blot. In the meantime, follicular fluid-derived granulosa samples were used as negative control. First, we compared the expression of CD63 and β-actin between FF-EVs and granulosa samples. The results disclosed that all expressions of β-actin were clearly probed in granulosa samples and were below the level of detection in FF-EVs samples, oppositely, CD63 exhibited clear bands in FF-EVs samples, but not probed in granulosa samples (Fig. 1D), indicating that the extracellular vesicles isolated from follicular fluid were not contaminated by follicular fluid-derived cells, such as granulosa. Next, all six samples were further validated for subsequent study. The results uncovered that TSG101 and CD63 presented obvious bands in all six samples (Fig. 1E), suggesting that FF-EVs were extracted successfully from 6 samples. Taken together, these data display that we successfully extracted FF-EVs that were not significantly different from other sources-derived extracellular vesicles in morphology.

**Table 1:** Clinical characteristics of PCOS and control subjects

Project	C1	C2	C3	P1	P2	P3	*p* value
Age (years)	28.5	27.3	29.2	25.3	26. 5	28.6	0.240209
BMI (kg/m^2^)	23.3	24.9	22.0	19.9	23.0	24.7	0.624407
LH (IU/L)	6.1	8.1	5.1	10.1	12.3	14.9	**0.0217718**
FSH (IU/L)	7.0	6.0	7.8	6.4	6.0	5.1	0.164435
E2 (pmol/L)	119.7	106.3	132.5	168.6	158.3	159.6	**0.00658136**
T (nmol/L)	1.0	0.5	1.3	3.5	2.9	2.3	**0.0092485**
FBG (mmol/L)	5.1	5.2	5.0	5.0	5.2	5.3	0.561437
Infertility (years)	3.9	3.6	4.1	2.9	2.6	3.5	**0.0454128**
PRL (ng/mL)	19.8	25.3	15.4	31.6	36.5	42.9	**0.0179693**
AMH (ng/mL)	4.1	5.6	6.8	9.2	11.9	13.5	**0.0150721**
Number of follicles	12	11	12	22	22	21	**<0.0001**

*BMI*, body mass index; LH, luteinizing hormone; *FSH*, follicle-stimulating hormone; *E2*, estradiol; *T*, testosterone; *FBG*, fasting blood glucose; *PRL*, prolactin; *AMH*, anti-Müllerian hormone. The bold numbers indicate statistically significant values

### Analysis of miRNAs in FF-EVs

**Fig. 2:**
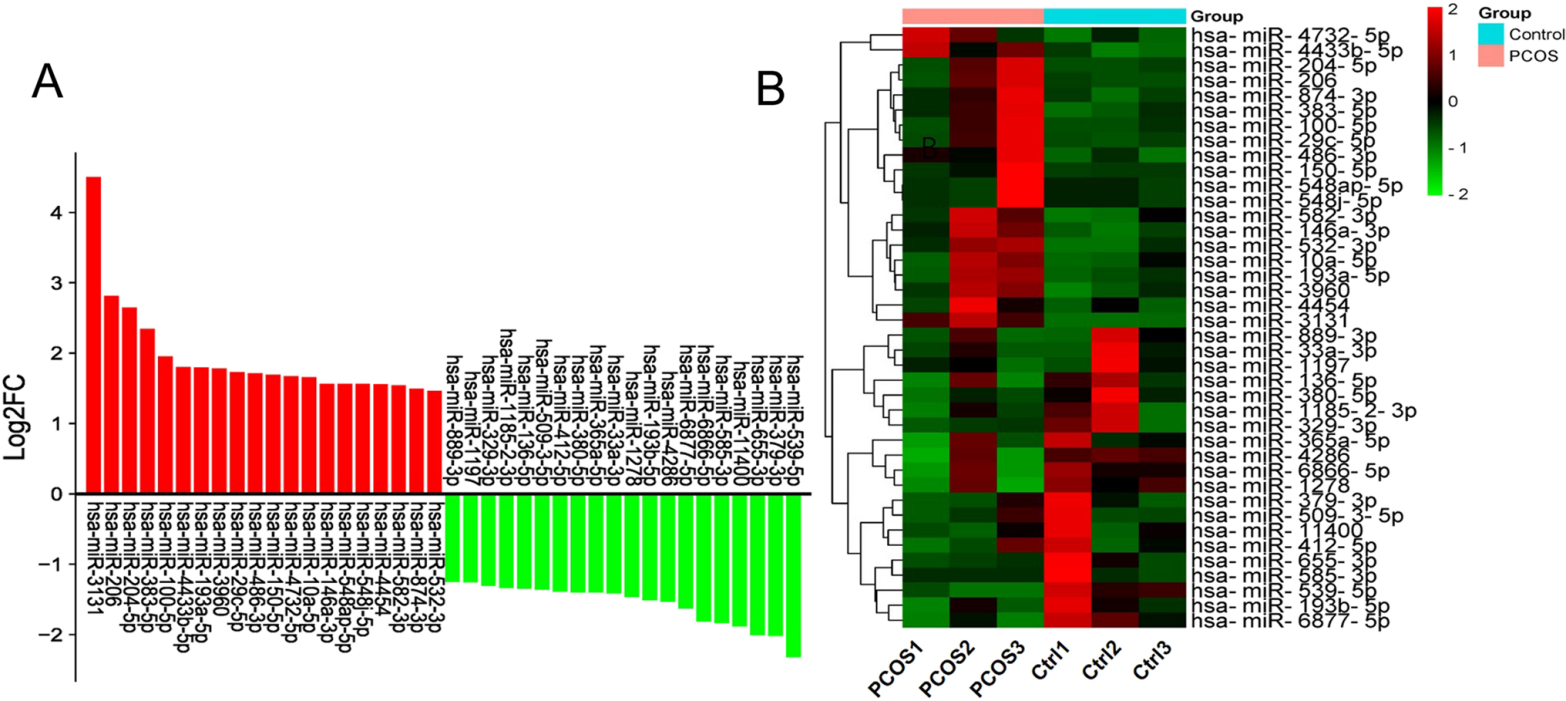
Comparison of expression levels of the up-regulated and down-regulated of top 20 DEmiRNAs in 6 FF-EVs samples. (**A**) Histogram of upregulated and downregulated of top 20 miRNAs. (**B**) Clustering analysis of upregulated and downregulated of top 20 miRNAs

To ascertain the main miRNAs contained in FF-EVs and involved in PCOS, high-throughput sequencing analysis was conducted. The results of sequencing revealed that many miRNAs were differentially expressed in FF-EVs of PCOS compared with control. A total of 1350 differentially expressed miRNAs (DEmiRNAs) from 6 samples of FF-EVs were identified. Of these DEmiRNAs, 747 were upregulated and 603 were downregulated. Moreover, based on the criteria of |log2FC| >1 and *p* ≤ 0.05, 514 DEmiRNAs were found to have significant changes, in which, 267 were upregulated and 247 were downregulated, and the top 20 most significantly upregulated and downregulated DEmiRNAs presented in a histogram (Fig. 2A) and Table 2. The 40 DEmiRNAs were selected for further clustering analysis. As seen in Fig. 2B, PCOS samples appeared distinctly separated from control samples.

**Table 2:** The top 20 up-and down-regulated miRNAs (the miRNA with statistical differences are bolded)

miRNA	Direction	Log2Foldchange	FDR	*p* value
**hsa-miR-3131**	**up**	**4.501**	**0.047**	**0.001**
**hsa-miR-206**	**up**	**2.813**	**0.95**	**0.015**
**hsa-miR-204-5p**	**up**	**2.645**	**0.55**	**0.005**
hsa-miR-383-5p	up	2.346	1	0.106
**hsa-miR-100-5p**	**up**	**1.955**	**0.93**	**0.014**
hsa-miR-4433b-5p	up	1.804	1	0.103
**hsa-miR-193a-5p**	**up**	**1.797**	**1**	**0.046**
hsa-miR-3960	up	1.780	1	0.120
hsa-miR-29c-5p	up	1.731	1	0.116
hsa-miR-486-3p	up	1.712	1	0.201
hsa-miR-150-5p	up	1.695	1	0.072
hsa-miR-4732-5p	up	1.671	1	0.261
hsa-miR-10a-5p	up	1.655	1	0.237
hsa-miR-146a-3p	up	1.565	1	0.211
hsa-miR-548ap-5p	up	1.561	1	0.227
hsa-miR-548j-5p	up	1.561	1	0.227
hsa-miR-4454	up	1.556	1	0.227
hsa-miR-582-3p	up	1.541	1	0.281
hsa-miR-874-3p	up	1.495	1	0.120
hsa-miR-532-3p	up	1.464	1	0.239
hsa-miR-889-3p	down	-1.250	1	0.183
hsa-miR-1197	down	-1.257	1	0.179
hsa-miR-329-3p	down	-1.306	1	0.168
hsa-miR-1185-2-3p	down	-1.332	1	0.226
hsa-miR-136-5p	down	-1.347	1	0.220
hsa-miR-509-3-5p	down	-1.359	1	0.328
hsa-miR-412-5p	down	-1.385	1	0.642
hsa-miR-380-5p	down	-1.397	1	0.205
hsa-miR-365a-5p	down	-1.398	1	0.346
hsa-miR-33a-3p	down	-1.412	1	0.258
hsa-miR-1278	down	-1.462	1	0.337
hsa-miR-193b-5p	down	-1.510	1	0.196
hsa-miR-4286	down	-1.531	1	0.262
hsa-miR-6877-5p	down	-1.626	1	0.401
hsa-miR-6866-5p	down	-1.812	1	0.328
hsa-miR-585-3p	down	-1.836	1	0.212
hsa-miR-11400	down	-1.882	1	0.147
hsa-miR-655-3p	down	-2.004	1	0.065
hsa-miR-379-3p	down	-2.015	1	0.327
**hsa-miR-539-5p**	**down**	**-2.319**	**1**	**0.050**

*FDR*, false discovery rate

**Fig. 3:**
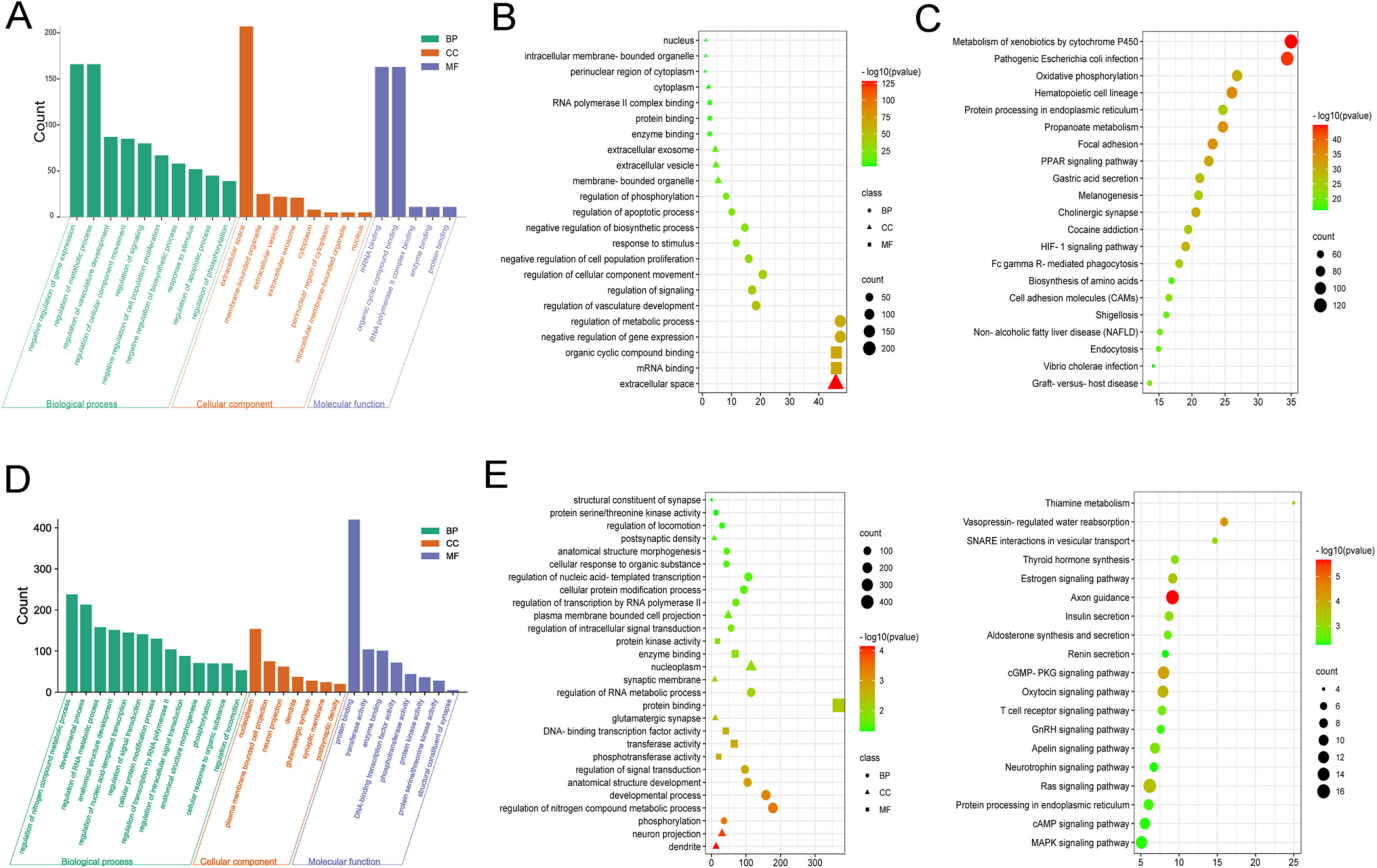
GO function annotation and KEGG pathways analysis of DEmiRNAs in FF-EVs. (**A**) The GO terms of 514 DEmiRNAs, including BP on the left, CC in the middle, and MF on the right. (**B**) Multi-class scatter plot of GO terms analysis of 514 DEmiRNAs. (**C**) Scatter plot of KEGG enrichment analysis of 514 DEmiRNAs. (**D**) The GO terms of 628 candidate predicted target genes of 6 DEmiRNAs, including BP on the left, CC in the middle, and MF on the right. (**E**) Multi-class scatter plot of GO terms analysis of 628 candidate predicted target genes of 6 DEmiRNAs. (**F**) Scatter plot of KEGG enrichment analysis of 628 candidate predicted target genes of 6 DEmiRNAs

Then, the total 514 DEmiRNAs were analyzed for GO terms and KEGG pathways. As shown in (Fig. 3A and B), the biological process domain mainly focused on negative regulation of gene expression and regulation of metabolic process. In the cellular component domain, it was mainly concentrated in the extracellular space and closely related to the membrane composition; in the molecular function domain, the mRNA binding and organic cyclic compound binding processes were enriched. In the enriched KEGG pathways (Fig. 3C), some pathways, such as metabolism of xenobiotics by cytochrome P450 and oxidative phosphorylation, were very significant, which has long been proven to play a very important role in the female reproductive system, especially in the occurrence of PCOS [27–31]. In the KEGG pathway, we also found that the pathogenic Escherichia coli infection pathway was significantly enriched in co-expressed miRNA target genes, indicating that extracellular vesicles may play a key role in the bacteriostatic process in follicular fluid.

Next, in order to further explore the physiological roles and regulatory mechanisms of DEmiRNAs in FF-EVs possibly involved in PCOS, 514 DEmiRNAs were narrowed down according to the highly significant *p* value. 6 DEmiRNAs, e.g., hsa-miR-3131(up), hsa-miR-206 (up), hsa-miR-204-5p (up), hsa-miR-100-5p (up), hsa-miR-193a-5p (up), and hsa-miR-539-5p (down), were picked out for further predicting their target genes. A total of 628 candidate target genes were predicted by miRWalk and subjected to GO annotation as well as KEGG analysis through GeneTrail 3.2. As shown in Fig. 3D, GO terms derived from the biological process mainly focused on metabolic processes, such as nitrogen compound metabolic process, RNA metabolic process, and some processes related to transcription. In the cellular component domain, the GO terms for the target genes included nucleoplasm, cell projection, and some pathways related to synapse. Molecular function mainly devoted to some processes such as protein binding and transferase and kinase activities. Multi-class scatter plot presented the results of GO terms more intuitively (Fig. 3E). In these enrichment terms, in addition to being closely related to some metabolic processes, many terms were enriched in signal transduction process, especially in some processes related to synapses, suggesting that DEmiRNAs in FF-EVs might be closely related to synapse-related signal transduction processes that is an important process of hormonal changes related to PCOS patients [32, 33]. Among the pathways enriched by KEGG (Fig. 3F), some metabolic pathways like insulin secretion and thiamine metabolism were enriched, and two hormone-related pathways—estrogen signaling pathway and GnRH signaling pathway—were also significantly enriched. In addition, Apelin signaling pathway was also captured. These differential miRNAs in FF-EVs of PCOS patients may have an impact on receptor cell-related metabolic processes, especially hormone metabolism-related processes.

**Fig. 4:**
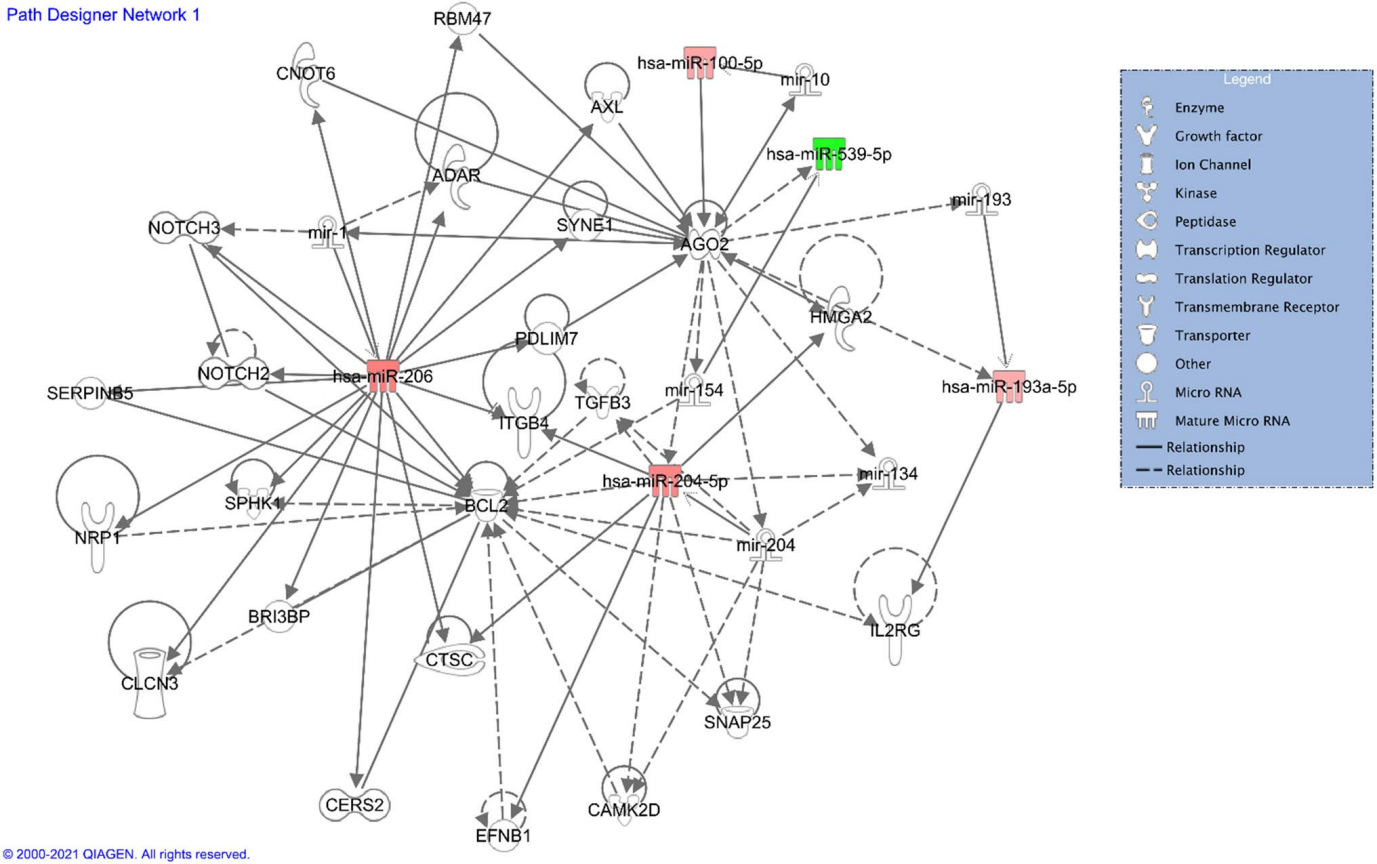
Ingenuity pathway analysis of DEmiRNAs in FF-EVs. Upregulated miRNAs are shown in red, and downregulated miRNAs are shown in green

Ingenuity pathway analysis (IPA) enables us to analyze and clarify the possible upstream regulator and downstream effects on cell and organism biology, as well as their interactions [34, 35]. Using ingenuity pathway assessment to analyze 6 DEmiRs, the results showed that, at the disease level, they are mainly related to body damage and abnormalities, reproductive system diseases, and cancer, and at the molecular and cellular functions level, they are mainly related to cell death and survival, cellular development, bio functions information for IPA analysis is in Supplementary file. Furthermore, based on the IPA network shown in Fig. 4, we could see that mir204-5p and mir-206 are closely related to BCL2, one of the anti-apoptotic genes. Therefore, it can be inferred that FEE-derived mir204-5p and mir-206 may be the key factors leading to the increase of follicles in patients with PCOS and by affecting the expression of BCL2 gene.

### Characterization and analysis of DEPs in FF-EVs

**Fig. 5:**
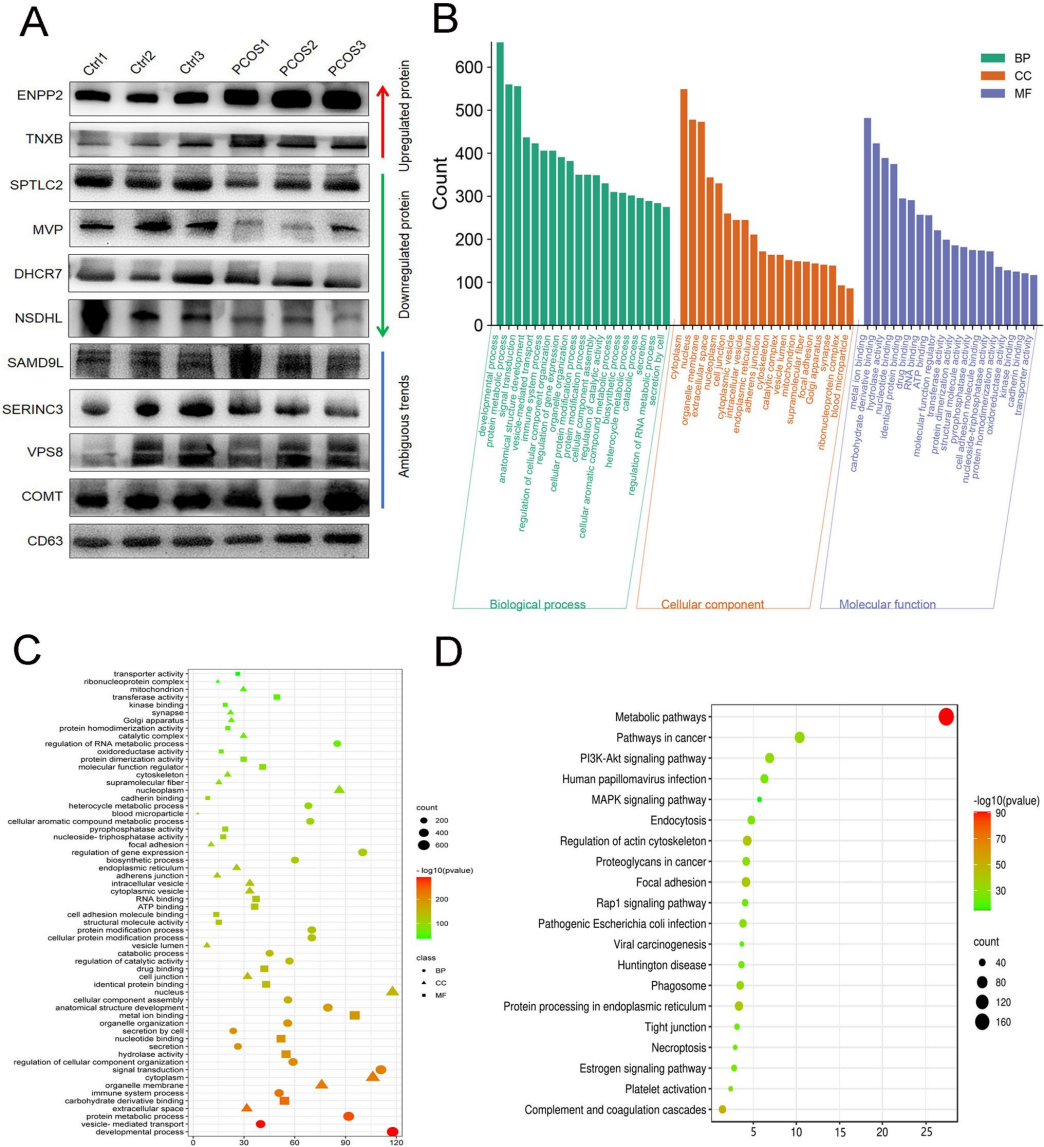
TMT assay validation and preliminary GO and KEGG analyses of 2487 DEPs in FF-EVs. (**A**) TMT assay validation by western blotting. (**B** and **C**) GO function annotation of 2487 DEPs. (**D**) Scatter plot of KEGG enrichment analysis of all quantifiable proteins

The protein components in FF-EVs from normal and PCOS patients were analyzed using TMT based quantitative proteomics system. A total of 3104 proteins were identified, in which, 2487 were quantifiable proteins (1051 upregulated and 1436 downregulated). 10 proteins (three upregulated proteins and seven downregulated proteins) were picked out to validate the TMT results by performing western blotting. Of ten proteins, two upregulated proteins (ENPP2 and TNXB) and downregulated proteins (SPTLC2, MVP, NSDLH, and DHCR7) were definitely confirmed by western blotting (Fig. 5A), and other four proteins, e.g., SAMD9L, SERINCE3, VPS8, and COMT, presented ambiguous trends, indicating that the TMT results were basically reliable. Subsequently, the identified 2487 quantifiable proteins were subjected to bioinformation analysis. GO terms showed that the biological process mainly involved in developmental process, protein metabolic process, signal transduction, and immune system process. In cellular component domain, cytoplasm, organelle membrane, extracellular space, and nucleoplasm were mainly enriched. Additionally, metal ion binding, carbohydrate derivative binding, hydrolase activity, and nucleotide binding were enriched in molecular function (Fig. 5B). GO terms were also intuitively showed by multi-class scatter plot (Fig. 5C). As shown in Fig. 5D, KEGG results showed that all quantifiable proteins were mainly enriched in metabolic pathways, pathways in cancer, PI3K-Akt signaling pathway, and regulation of actin cytoskeleton. And HPV infection and the pathogenic E. coli infection pathway were also significantly enriched, further indicating that FF-EVs may play an anti-inflammatory and bacteriostatic effect in follicles.

**Fig. 6:**
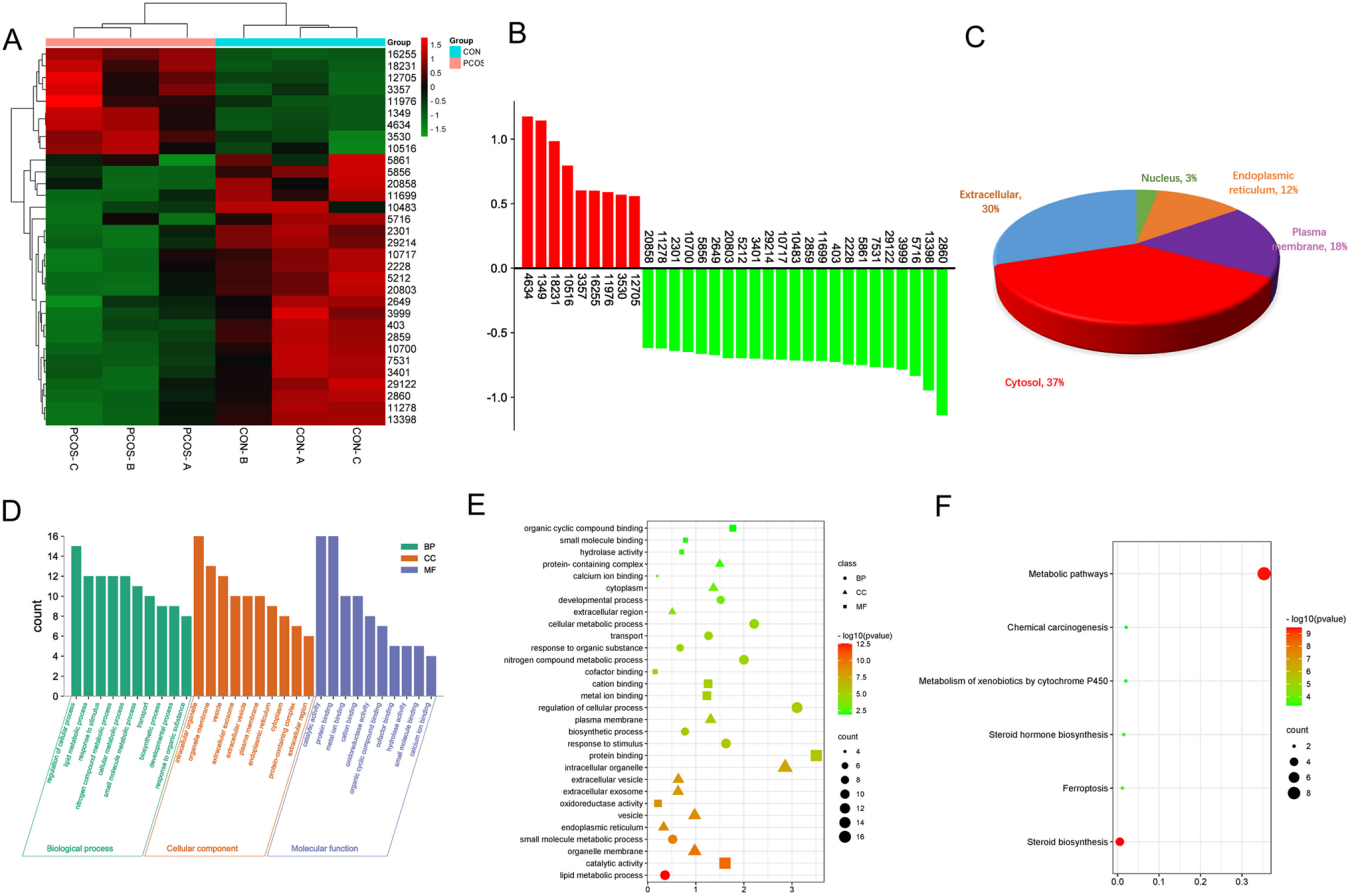
Comparison of the expression levels of the highly enriched 32 DEPs in FF-EVs in 6 FF-EVs samples and their subcellular localization. (**A**) Heatmap and Clustering of 32 DEPs in FF-EVs. (**B**) Histogram of 32 DEPs in FF-EVs. (**C**) Subcellular localization of 32 DEPs in FF-EVs. (**D** and **E**) The GO terms of DEPs include BP, CC, and MF. (**F**) Scatter plot of KEGG enrichment analysis of 32 DEPs in FF-EVs

To further narrow down the target proteins, according to the protein ratio, the *t*-test was performed, and the *p* value was calculated as a significant index. Finally, we found that there were 32 significant differentially expressed proteins (DEPs) between PCOS and normal controls. Among them, 9 proteins in PCOS patients were significantly higher than that in control group, and 23 proteins were lower than that in control group. The cluster heat map of the above 32 DEPs was shown in Fig. 6A, and the histogram showed the expression levels of these proteins (Fig. 6B). The information of DEPs was listed in Table 3. The subcellular localization analysis of 32 DEPs indicated that most proteins were located in cytoplasm, extracellular, and membrane organelles (Fig. 6C). The identified 32 DEPs were performed GO functional annotation and KEGG pathway analysis using the Gene Trail website tool. In the biological process, we could see that the DEPs mainly participated in various metabolic processes, i.e., lipid metabolic process and nitrogen compound metabolic process, and these metabolic processes have long been proved to be closely related to PCOS [36–39]. In the cellular component domain, the differential proteins were mainly related to the processes of intracellular organelle, organelle membrane, and vesicle. In the terms of molecular function, the oxidoreductase activity pathway was significantly enriched (Fig. 6D and E), which has long been proved to be closely related to the pathology and pathophysiology of PCOS [27, 30, 31, 40].

**Table 3:** DEPs in FF-EVs of PCOS and normal controls

UniProtKB-AC		Protein name	Foldchange	FDR	*p* value
P28161	GSTM2	Glutathione S-transferase Mu 2	2.258	0.96	0.011
Q8IVG5	SAMD9L	Sterile alpha motif domain-containing protein 9-like	2.209	0.96	0.011
Q9BYE9	CDHR2	Cadherin-related family member 2	1.977	0.96	0.004
P35542	SAA4	Serum amyloid A-4 protein	1.734	0.99	0.047
Q13822	ENPP2	Ectonucleotide pyrophosphatase/phosphodiesterase family member 2	1.516	0.96	0.019
O95932	TGM3L	Protein-glutamine gamma-glutamyltransferase 6	1.514	0.47	0.000
P22105	TENX	Tenascin-X	1.503	0.99	0.048
P00748	FA12	Coagulation factor XII	1.483	0.96	0.009
O95497	VNN1	Pantetheinase	1.471	0.96	0.023
Q15043	S39AE	Zinc transporter ZIP14	0.651	0.97	0.035
O15270	SPTC2	Serine palmitoyltransferase 2	0.649	0.96	0.026
O75976	CBPD	Carboxypeptidase D	0.641	0.96	0.006
O75396	SC22B	Vesicle-trafficking protein SEC22b	0.637	0.96	0.013
P0DOY2	IGLC2	Immunoglobulin lambda constant 2	0.630	0.96	0.011
Q16850	CP51A	Lanosterol 14-alpha demethylase	0.627	0.98	0.045
Q96K37	S35E1	Solute carrier family 35 member E1	0.616	0.96	0.021
P37058	DHB3	Testosterone 17-beta-dehydrogenase 3	0.616	0.96	0.023
P07099	HYEP	Epoxide hydrolase 1	0.614	0.96	0.018
Q3KR37	GRAMD1B	GRAM domain-containing protein 1B	0.612	0.90	0.003
Q9UBV2	SE1L1	Protein sel-1 homolog 1 or Suppressor of lin-12-like protein	0.611	0.98	0.044
P21817	RYR1	Ryanodine receptor 1 or Skeletal muscle calcium release channel	0.608	0.98	0.041
Q15392	DHC24	Delta(24)-sterol reductase	0.607	0.96	0.004
Q13530	SERC3	Serine incorporator 3	0.606	0.96	0.014
P51648	AL3A2	Fatty aldehyde dehydrogenase	0.604	0.96	0.007
P21964	COMT	Catechol O-methyltransferase	0.595	0.96	0.026
A0M8Q6	IGLC7	Immunoglobulin lambda constant 7	0.594	0.97	0.028
Q14764	MVP	Major vault protein	0.587	0.96	0.019
Q8N3P4	VPS8	Vacuolar protein sorting-associated protein 8 homolog	0.586	0.97	0.033
P02792	FRIL	Ferritin light chain	0.579	0.97	0.030
P01834	IGKC	Immunoglobulin kappa constant	0.560	0.98	0.039
Q15738	NSDHL	Sterol-4-alpha-carboxylate 3-dehydrogenase, decarboxylating	0.518	0.96	0.020
Q9UBM7	DHCR7	7-Dehydrocholesterol reductase	0.43	0.96	0.024

*FDR*, false discovery rate

KEGG pathway analysis also revealed 6 pathways with high enrichment of DEPs, and these pathways were mainly involved in metabolic pathway, metabolism of xenobiotics by cytochrome P450, steroid hormone biosynthesis, steroid biosynthesis, and ferroptosis (Fig. 6F), which have been proven to be related to the occurrence of PCOS [27–29].

### Possible interaction of DEPs in FF-EVs

**Fig. 7:**
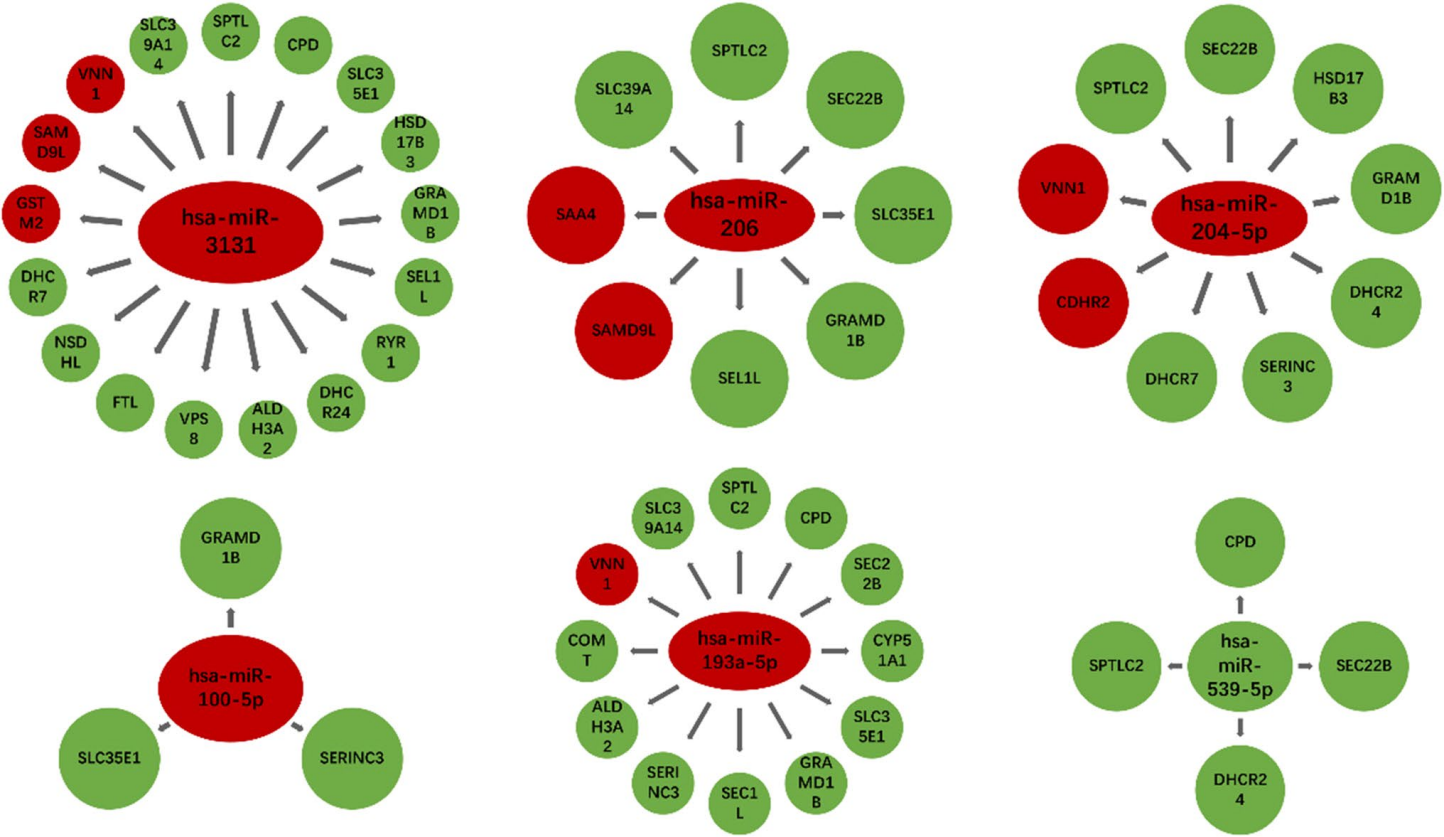
Correlation diagram of DEmiRs target genes and DEPs. There was a corresponding relationship between the target gens of 6 differential miRNAs and DEPs. The red and green color represented upregulation and downregulation DEPs, respectively

As shown in Fig. 7, the correlation diagrams were made by integrating the 6 DEmiRNA target gene data sets and DEPs data sets. We found that there was a corresponding relationship between the predicted target genes of 6 differential miRNAs and DEPs. Has-miR-3131’s target genes were related to 17 differential proteins, the target genes of has-miR-204-5p matched 9 DEPs, has-miR-206 matched 8 DEPs, has-miR-193a-5p matched 12 DEPs, and has-miR-100-5p involved in 3 DEPs. In addition, there were 4 DEPs associated with has-miR-539-5p. The red and green color represented upregulation and downregulation DEPs, respectively. Especially, GRAMD1B protein corresponded to all 5 upregulated DEmiRNAs, and SPTLC2 protein corresponded to 4 upregulated and one downregulated miRNAs. Collectively, by integrating DEmiRNAs and DEPs analyses, GRAMD1B and SPTLC2 were considered to be critical molecules involving in PCOS.

## Discussion

The characteristics of extracellular vesicles are as described previously. The extracellular vesicles in ovarian follicular fluid are involved in the exchange of genetic information between cells. Thus, extracellular vesicles are often used as indicators of oocyte quality and competence [41]. Although some studies reported the related substances in the FF-EVs of PCOS patients, such as miRNA, lncRNA, or protein, there were few studies on both miRNA and protein levels in FF-EVs in PCOS patients. Moreover, their potential interactions are not yet clarified.

In the present study, we obtained the miRNA and protein expression profiles of FF-EVs and further comprehensively analyzed the potential role of miRNAs and proteins in PCOS. Through further biometric analysis, we found that 6 miRNAs and 32 proteins were significantly differentially expressed between PCOS and the control group.

In analyzing the DEmiRNA target genes, GnRH signaling pathway was noted. In line with our analysis, previous research has verified GnRH is closely related to PCOS by regulating neurotransmitters [42].

Interestingly, Apelin signaling pathway was caught in our analysis. In keeping with our results, Apelin has been closely associated with PCOS based on the following facts. Studies have shown that Apelin/apelin-receptor also expressed in ovary such as follicles and granulosa cells, indicating that the Apelin/apelin-receptor plays an important role in the development of follicle. Furthermore, Apelin/apelin-receptor plays roles in vascular establishment and hormone metabolism in ovary. Increased Apelin/apelin-receptor expression has been found in ovary of PCOS, which are associated with abnormal ovarian hormones and function [43, 44].

Considering the fact that determining the vital genes or molecular details involving in PCOS by using miRNAs or proteins expression data alone is very limited and may not be sufficient [45], we therefore integrated miRNA and protein expression data in order to identify PCOS-related miRNAs and investigated relationships between miRNAs and the regulatory networks in PCOS. By integrating the corresponding relationship between the target gene results predicted by the miRWalk database and the differential proteins, it was found that some differential proteins can correspond to the target gens of differential miRNAs. In integrating analyses, we noticed that the enriched pathways in DEmiRNAs target genes and DEPs data sets are very similar, e.g., hormone-related metabolic processes.

**Fig. 8:**
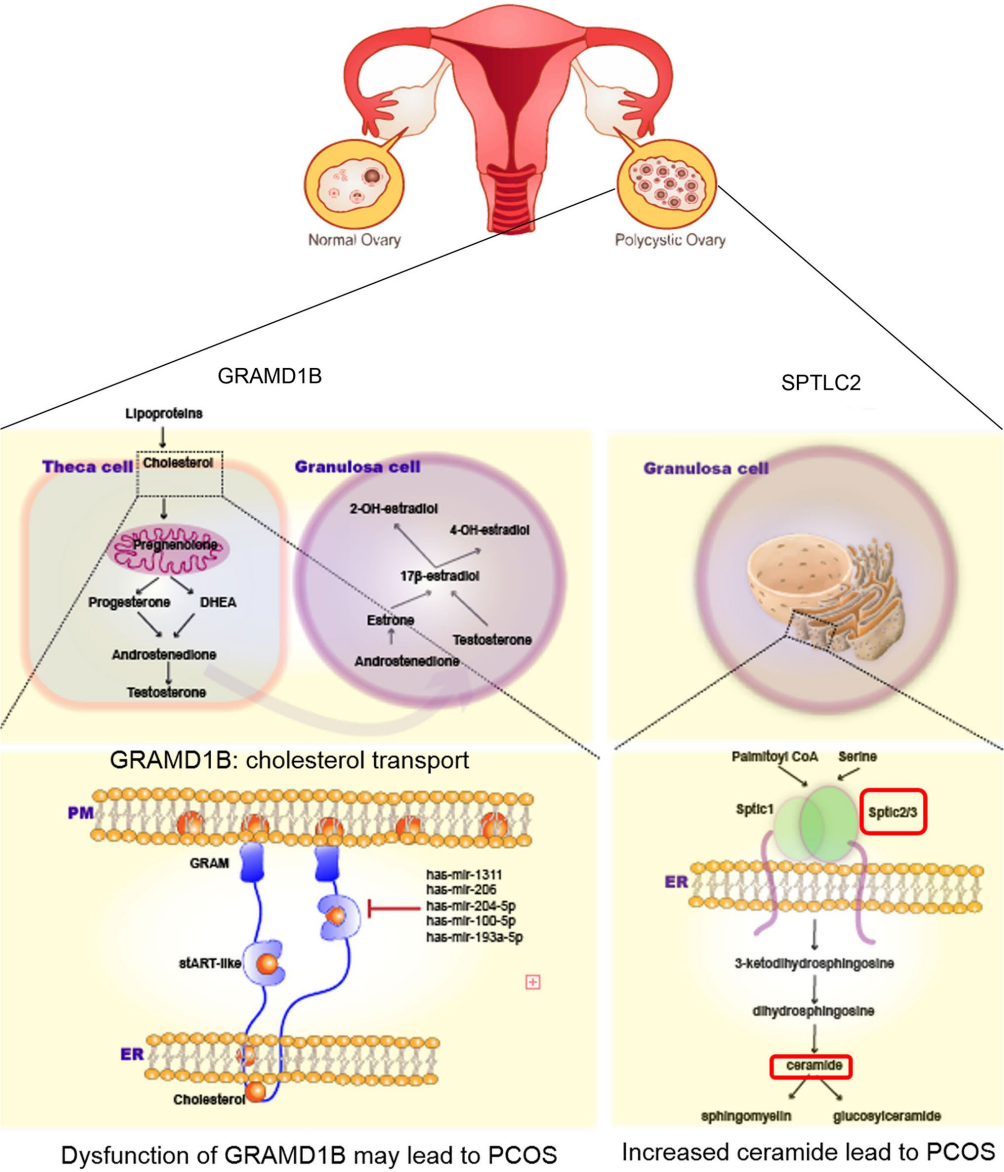
Possible molecular mechanisms of GRAMD1B and SPTLC2 involving in PCOS. (**A**) GRAMD1s comprise an N-terminal GRAM domain and a StART-like domain, which is followed by a C-terminal transmembrane domain that anchors the protein to the ER. GRAMD1s move to ER-PM (plasma membrane) contacts by sensing accessible PM cholesterol via the GRAM domain and can transport accessible cholesterol from the PM to the ER via the StART-like domain. GRAMD1s family are important for cholesterol homeostasis. GRAMD1B protein presented a downregulated level in FF-EVs of PCOS patients. Simultaneously, *gramd1b* gene is the predicted targets of 5 DEmiRNAs, including has-mir-3131, has-mir-206, has-mir-204-5p, has-mir-100-5p, and has-mir-193a-5p, found to be upregulated in FF-EVs of PCOS patients. An imagined work scenario should be decreased GRAMD1 expression or loss of GRAMD1 function caused by upregulated miRNAs led to sustained accumulation of accessible cholesterol in the PM and possible dysregulation of cellular cholesterol homeostasis as well as altered steroid hormone production and activity, eventually resulting in POCS. (**B**) SPT is a rate-limiting step in the de novo ceramide biosynthesis pathway and is composed of two main subunits, namely, Sptlc1 and Sptlc2. Increased ceramide subclasses have also been identified as novel lipidomic biomarkers in PCOS. High ceramide levels have been consistently linked with insulin resistance and the development of diabetes, suggesting that SPTLC2 is the key hub in PCOS

Two important molecules, GRAMD1B and SPTLC2, should be emphasized. First, to the best of our knowledge, hyperandrogenism which involves high overall levels of circulating testosterone is one of the important criteria for diagnosing PCOS, and testosterone is a steroid hormone which is directly metabolized from cholesterol. Specifically, the latest study revealed that GRAMD1s belong to endoplasmic reticulum (ER)-anchored protein that is expressed in eukaryotic cells. GRAMD1s comprise an N-terminal GRAM domain and a StART-like domain, which is followed by a C-terminal transmembrane domain that anchors the protein to the ER [46, 47]. They move to ER-PM (plasma membrane) contacts by sensing accessible PM cholesterol via the GRAM domain and can transport accessible cholesterol from the PM to the ER via the StART-like domain. Therefore, the members of GRAMD1s family are important for cholesterol homeostasis. The GRAMD1b GRAM domain possesses distinct sites to detect accessible cholesterol and anionic lipids within the PM [48]. Up to now, there is no relevant study on the involvement of GRAMD1B in PCOS. In present study, we have noted that GRAMD1B protein presented a downregulated level in FF-EVs of PCOS patients. Simultaneously, very importantly, *gramd1b* gene is the predicted targets of 5 DEmiRNAs that were found to be upregulated in FF-EVs of PCOS patients. We therefore imagined a work scenario, in which decreased GRAMD1 expression or loss of GRAMD1 function caused by upregulated miRNAs (including has-mir-3131, has-mir-206, has-mir-204-5p, has-mir-100-5p, and has-mir-193a-5p) led to sustained accumulation of accessible cholesterol in the PM and possible dysregulation of cellular cholesterol homeostasis as well as altered steroid hormone production and activity, eventually resulting in POCS (Fig. 8A).

Second, ceramide is a sphingolipid metabolite and a major component of the plasma membrane and lipoproteins. De novo synthesis of ceramide starts from the condensation of serine and palmitoyl CoA. Fatty acids (FAs) are converted to ceramide via a series of reactions by serine palmitoyltransferase (SPT), 3-ketosphinganine reductase, ceramide synthase, and dihydroceramide desaturase. SPT is a rate-limiting step in the de novo ceramide biosynthesis pathway and is composed of two main subunits, namely, Sptlc1 and Sptlc2 [49]. The latter encodes a long chain base subunit of serine palmitoyltransferase. Each subunit is stabilized by forming a dimer or multimer in the endoplasmic reticulum to produce ceramide [50]. Increased ceramide subclasses have also been identified as novel lipidomic biomarkers in PCOS [51]. In addition, PCOS is also closely associated with insulin resistance and is linked to an increased risk of developing type 2 diabetes [52]; meanwhile, high ceramide levels have been consistently linked with insulin resistance and the development of diabetes [53], suggesting that SPTLC2 is the key hub in PCOS (Fig. 8B). Our results showed the decreased expression of SPTLC2 in FF-EVs of PCOS patients. These evidences suggest that GRAMD1B and SPTLC2 play vital roles in PCOS. More in-depth molecular mechanisms need to be further explored.

It should be also addressed that recent research found that downregulation of miR-206 can promote the human granulosa-like tumor cell line, KGN cells, proliferation, inhibit cell apoptosis, and further promote the development of PCOS [54, 55]. Jiang et al. found that high expression of miR-204 can improve insulin resistance (IR) of PCOS via the inactivation of TLR4/NF-κB pathway [56]. In our results, miR-206 and miR-204-5p were also downregulated, and by IPA analysis, it was further found that these two miRNAs were both positively related to the anti-apoptotic factor BCL2 gene. Previous study showed that an increase in ovarian apoptosis caused by an imbalance among the Bcl-2 family members may be involved in the transformation of growing follicles in cystic follicles in the ovaries from DHEA-induced PCOS rats [57]. Thereby, miR-206 and miR-204-5p play important roles in PCOS. Several DEPs found in our study have also been reported in previous studies. For instance, Trine Maxel recently found that zip14 (SLC39A14) may affect zinc homeostasis in adipose tissue in PCOS patients [58]. Insulin receptor substrate-related serine phosphorylation affects the metabolism of classical insulin target tissues, which may be the mechanism of PCOS defect after the characteristic combination of insulin action [59, 60]. Wehr and colleagues found that related polymorphisms of the DHCR7 gene are associated with insulin resistance and vitamin D deficiency in PCOS [61]. Our results showed that the expression status of these key proteins in the FF-EVs of PCOS patients is also different, indicating that these proteins may play relevant roles through FF-EVs in vivo.

Herein, it deserves to be addressed that having hemorrhage is very common after puncturing the first follicles and due to the high number of follicles in PCOS patients. The samples used in this study did not contain blood because FFs were only collected when they were clear with the naked eye. In this way, even if the samples were contaminated with blood, we believed that its amount was very little, and its impact on the whole follicular fluid should be generally ignored.

In summary, the results give us a better understanding of intra-follicular abnormalities in PCOS. And our research shows that, compared with the control group, various miRNAs and proteins in PCOS female FF-EVs are differentially expressed, some of which are important in hormone metabolism pathways. In addition, the pathways of miRNA and protein enrichment in FF-EVs are similar, and there is a corresponding relationship between DEmiR and DEPs, illustrating that there may be regulatory relationships between miRNAs and proteins in FF-EVs. However, regarding the specific interactions between miRNAs and proteins in FEEs and the mechanism of action of different miRNAs and proteins in driving the occurrence of PCOS, many questions have not yet been answered, so further research is needed.

## Material and methods

### Follicular fluid (FF) sample collection

The patients who donated the FF samples used in this study were undergoing routine *in vitro* fertilization (IVF) treatment. In addition, all experiments were approved by the ethics committee of Nanjing Maternity and Child Health Care Hospital (NJFY-2019KY-020). An informed consent form has been obtained from each couple regarding the use of FF samples obtained during IVF treatment for this study. The current diagnostic criterion for PCOS is based on the revised 2003 criteria (two out of three is enough for positive diagnosis) as follows: (a) oligo-ovulation and/or anovulation, (b) clinical and/or biochemical signs of hyperandrogenism;, and (c) polycystic ovaries. The control group contained patients undergoing IVF due to male factor infertility or tubal factors. The exclusion criteria for the two groups included women with endometriosis, cancer, primary ovarian insufficiency (POI), or other medical diseases that may affect follicular development.

Control and PCOS patients (Table 1 contains all basic information of patients) received recombinant follicle-stimulating hormone (FSH) injection after treatment with GnRH agonists according to the standard regimen of IVF treatment. FSH stimulation was initiated once downregulation was confirmed by ultrasound and measurements of serum estradiol, luteinizing hormone, and progesterone. From the 5th day of FSH treatment to the day of egg retrieval, real-time ultrasound scans were used every two days to assess the growth of the follicles. When at least one follicle grew to 18–20 mm in diameter, the egg was punctured 34–38 h after hCG was triggered under the guidance of vaginal ultrasound. The sample was centrifuged at 3000 g for 15 minutes to remove cell debris and other particles. The supernatant was stored at −80 °C for future use.

### Exosome isolation

Follicular fluid extracellular vesicles (FF-EVs) were purified and characterized according to previously published protocols with some modifications [62, 63]. In detail, a total of 15 mL of pooled follicular fluid from the patient was obtained and was centrifuged at 3,500 rpm for 15 minutes at 4 °C to settle the debris. Then, the supernatants were transferred into a 15 mL ultracentrifuge tube, ultracentrifuged at 16,500 g for 30 minutes at 4 °C, and then filtered through a 0.2 mm syringe filter to obtain medium containing extracellular vesicles. Finally, the extracellular vesicles were pelleted by ultracentrifugation at 120,000 g for 70 minutes at 4 °C and stored at −80 °C for further analysis.

### Transmission electron microscopy

Extracellular vesicles were analyzed by transmission electron microscopy (TEM) as previously described [64, 65]. A total of 20 μL of exosome suspension (5 μg/μL) was fixed on a continuous grid and then negatively stained with 2% uranyl acetate solution for 1 minute and air-dried. The samples were observed by FEI Tecnai G2 spirit transmission electron microscope (FEITM) at an acceleration voltage of 120 kV.

### Nanoparticle tracking analysis

Nanoparticle tracking analysis (NTA) measurements were performed using a NanoSight NS300 instrument (Malvern Panalytical) with a 488-nm laser and sCMOS camera module (Malvern Panalytical). Measurements in flow mode were performed with a flow rate of 50, these flow measurements consisted of 3 measurements of 60 seconds, and the captured data were analyzed using NTA 3.2 software.

### Extraction of miRNA and protein from extracellular vesicles

Extracellular vesicle miRNAs were extracted from the aforementioned follicular fluid extracellular vesicles pellets using TruSeq Small RNA Sample Preparation kit (Illumina). The concentration of extracted protein was determined by Bradford method, iTRAQ® Reagent - 8PLEX Multiplex Kit (Sgima) was used for proteolysis, and iTRAQ® Reagent - Multiplex Buffer Kit (Sgima) was used for iTRAQ labeling.

### Western blot

Western blot assay was carried out as follows: extracellular vesicles were lysed with RIPA buffer (Santa Cruz, USA) and cleared lysate was collected by centrifugation for protein separation on 10% SDS polyacrylamide gel. The proteins were transferred onto PVDF membranes (Millipore) and detected with respective antibodies at 4 °C overnight, followed by incubation with IRDye Fluor 680-labeled IgG secondary antibody (Li-Cor Bioscience). The images were scanned and quantified by densitometric analysis by Li-COR Odyssey Infrared Imager. Primary antibodies against CD63 (Abcam) and TSG101(Proteintech) were used.

### miRNA analysis method

Reference genes and genome annotation files were downloaded from the ENSEMBL website (http://www.ensembl.org/ index.html). Bowtie was used to build a reference genome. Then, comparison of the clean data to the reference genome was carried out through Bowtie. TPM (Transcripts Per Million) represents the expression level of miRNA [66], based on the reads (not fully mature or degraded) that are compared to the miRNA precursor and slide in a certain area of the mature body.

### Protein analysis method

The protein samples were specifically labeled with TMT technology. This technology uses 6 or 10 isotopic labels to label the amino groups of polypeptides specifically. Then, to compare the relative or absolute content of proteins, tandem mass spectrometry analysis was performed. The protein sequences corresponding to the Homo sapiens protein library were identified in uniport (https://www.uniprot.org/proteomes/UP000005640) [67]. The pQuantMS2 in pFind Studio was used to perform quantitative calculations for TMT/iTRAQ. First, the ratio of each marker pair of each PSM was calculated, then the median of the ratio of the peptides contained in each protein was took, and the result was the corresponding protein. The ratio of marker was paired. Finally, the median of the ratio of all the biological replicates of the different samples was compared and the median was to be as the multiple of the difference between the final samples [68].

### Comprehensive bioinformatics analysis

The target genes of these six miRNAs were predicted by using the miRWalk database (http://mirwalk.umm.uni-heidelberg.de/) according to the base-pairing complementarity between the critical “seed” region of the mature miRNA (nt 2–7) and the 3′-UTR of a target genes mRNA, and the genes with high scores were further validated in the miRDB database and were selected. The Gene Ontology and Kyoto Encyclopedia of Genes and Genomes enrichment analysis were performed by using the online software (https://genetrail.bioinf.uni-sb.de/). The relevant results were visualized by using R language related package. The IPA system (version 62089861, Ingenuity Systems; Qiagen China Co., Ltd.) was used for subsequent bioinformatics analysis. GENEMANIA (http://genemania.org/search/) was used to construct a protein–protein interaction network for DEPs to evaluate the functions of these proteins. The associations between DEmiRNAs and DEPs were preliminary explored by integrating the intersection of miRNAs target genes predicted by miRWalk and differentially expressed proteins.

### Statistical analyses

Differential expression analysis of miRNAs in the two groups of samples was performed using DESeq in the R language package. miRNAs with *p* < 0.05 and |log2_ratio| ≥ 1 are identified as differentially expressed miRNAs [69]. The *t*-test was performed by using the protein ratio, and the *p* value was calculated as a significant index. The criteria for a significant increase were log2(Foldchange) > median + 2*s.iqr, and *p* value <0.05 was upregulated differential protein. The standard for significant downregulation was log2(Foldchange) < median-2*s.iqr, and *p* value <0.05 is the downregulation of differential protein [70].

## Data Availability

All data and materials generated for this study are available within the article and its supplementary materials.

## References

[1] Escobar-Morreale HF (2018). Polycystic ovary syndrome: definition, aetiology, diagnosis and treatment. Nat Rev Endocrinol.

[2] Azziz R (2016). Polycystic ovary syndrome. Nat Rev Dis Primers.

[3] Broekmans FJ (2006). PCOS according to the Rotterdam consensus criteria: change in prevalence among WHO-II anovulation and association with metabolic factors. Bjog.

[4] Duncan WC (2014). A guide to understanding polycystic ovary syndrome (PCOS). J Fam Plann Reprod Health Care.

[5] Yildiz BO (2012). Prevalence, phenotype and cardiometabolic risk of polycystic ovary syndrome under different diagnostic criteria. Hum Reprod.

[6] Azziz R (2004). The prevalence and features of the polycystic ovary syndrome in an unselected population. J Clin Endocrinol Metab.

[7] De Geyter C (2019). Assisted reproductive technology: impact on society and need for surveillance. Best Pract Res Clin Endocrinol Metab.

[8] Yin B (2015). Patients with polycystic ovary syndrome have successful embryo arrest. Int J Clin Exp Med.

[9] Rodgers RJ, Irving-Rodgers HF (2010). Formation of the ovarian follicular antrum and follicular fluid. Biol Reprod.

[10] Gosden RG (1988). Physiological factors underlying the formation of ovarian follicular fluid. J Reprod Fertil.

[11] Vlassov AV (2012). Exosomes: current knowledge of their composition, biological functions, and diagnostic and therapeutic potentials. Biochim Biophys Acta.

[12] Wang LP, et al. High throughput circRNAs sequencing profile of follicle fluid exosomes of polycystic ovary syndrome patients. J Cell Physiol. 2019. 10.1002/jcp.28201 10.1002/jcp.28201 30779115

[13] Tkach M, Théry C (2016). Communication by extracellular vesicles: where we are and where we need to go. Cell.

[14] Li S (2018). exoRBase: a database of circRNA, lncRNA and mRNA in human blood exosomes. Nucleic Acids Res.

[15] El Andaloussi S (2013). Extracellular vesicles: biology and emerging therapeutic opportunities. Nat Rev Drug Discov.

[16] Di Pietro C (2016). Exosome-mediated communication in the ovarian follicle. J Assist Reprod Genet.

[17] Hung WT (2015). Extracellular vesicles from bovine follicular fluid support cumulus expansion. Biol Reprod.

[18] Huang X (2020). Depletion of exosomal circLDLR in follicle fluid derepresses miR-1294 function and inhibits estradiol production via CYP19A1 in polycystic ovary syndrome. Aging.

[19] Yuan D (2021). PCOS follicular fluid derived exosomal miR-424-5p induces granulosa cells senescence by targeting CDCA4 expression. Cell Signal.

[20] Wang L (2020). Aberrant expression of long non-coding RNAs in exosomes in follicle fluid from PCOS patients. Front Genet.

[21] da Silveira JC (2012). Cell-secreted vesicles in equine ovarian follicular fluid contain miRNAs and proteins: a possible new form of cell communication within the ovarian follicle. Biol Reprod.

[22] Sohel MM (2013). Exosomal and non-exosomal transport of extra-cellular microRNAs in follicular fluid: implications for bovine oocyte developmental competence. PLoS One.

[23] Li H (2020). S100-A9 protein in exosomes derived from follicular fluid promotes inflammation via activation of NF-κB pathway in polycystic ovary syndrome. J Cell Mol Med.

[24] Krol J, Loedige I, Filipowicz W (2010). The widespread regulation of microRNA biogenesis, function and decay. Nat Rev Genet.

[25] Fabian MR, Sonenberg N, Filipowicz W (2010). Regulation of mRNA translation and stability by microRNAs. Annu Rev Biochem.

[26] Check Hayden E (2008). Thousands of proteins affected by miRNAs. Nature.

[27] Zhu W (2019). Oxidative stress increases the 17,20-lyase-catalyzing activity of adrenal P450c17 through p38α in the development of hyperandrogenism. Mol Cell Endocrinol.

[28] Grossman MP (2008). Müllerian-inhibiting substance inhibits cytochrome P450 aromatase activity in human granulosa lutein cell culture. Fertil Steril.

[29] Gray SA, Mannan MA, O'Shaughnessy PJ (1995). Development of cytochrome P450 aromatase mRNA levels and enzyme activity in ovaries of normal and hypogonadal (hpg) mice. J Mol Endocrinol.

[30] Agarwal A (2012). The effects of oxidative stress on female reproduction: a review. Reprod Biol Endocrinol.

[31] Bannigida DM, Nayak BS, Vijayaraghavan R (2020). Insulin resistance and oxidative marker in women with PCOS. Arch Physiol Biochem.

[32] Berg T, Silveira MA, Moenter SM (2018). Prepubertal development of GABAergic transmission to gonadotropin-releasing hormone (GnRH) neurons and postsynaptic response are altered by prenatal androgenization. J Neurosci.

[33] Porter DT (2019). Prenatal testosterone exposure alters GABAergic synaptic inputs to GnRH and KNDy neurons in a sheep model of polycystic ovarian syndrome. Endocrinology.

[34] Krämer A (2014). Causal analysis approaches in ingenuity pathway analysis. Bioinformatics.

[35] Liu CH, Di YP (2020). Analysis of RNA sequencing data using CLC genomics workbench. Methods Mol Biol.

[36] De Leo V (2016). Genetic, hormonal and metabolic aspects of PCOS: an update. Reprod Biol Endocrinol.

[37] Li Y (2019). Multi-system reproductive metabolic disorder: significance for the pathogenesis and therapy of polycystic ovary syndrome (PCOS). Life Sci.

[38] Polyzos SA (2018). Irisin in metabolic diseases. Endocrine.

[39] Liu Q (2019). Dyslipidemia involvement in the development of polycystic ovary syndrome. Taiwan J Obstet Gynecol.

[40] Peng Y (2020). Novel mechanisms underlying anti-polycystic ovary like syndrome effects of electroacupuncture in rats: suppressing SREBP1 to mitigate insulin resistance, mitochondrial dysfunction and oxidative stress. Biol Res.

[41] Revelli A (2009). Follicular fluid content and oocyte quality: from single biochemical markers to metabolomics. Reprod Biol Endocrinol.

[42] Chaudhari N, Dawalbhakta M, Nampoothiri L (2018). GnRH dysregulation in polycystic ovarian syndrome (PCOS) is a manifestation of an altered neurotransmitter profile. Reprod Biol Endocrinol.

[43] Liu Q (2020). Apelin/Apelin receptor: a new therapeutic target in polycystic ovary syndrome. Life Sci.

[44] Kurowska P (2018). Apelin in reproductive physiology and pathology of different species: a critical review. Int J Endocrinol.

[45] Schwanhausser B (2013). Corrigendum: global quantification of mammalian gene expression control. Nature.

[46] Besprozvannaya M, et al. GRAM domain proteins specialize functionally distinct ER-PM contact sites in human cells. Elife. 2018:7. 10.7554/eLife.31019 PMC5823543 29469807

[47] Nunes P, Demaurex N (2018). GRAM marks the spot for STIM. Commentary on "GRAM domain proteins specialize functionally distinct ER-PM contact sites in human cells". Cell Calcium.

[48] Sandhu J (2018). Aster proteins facilitate nonvesicular plasma membrane to ER cholesterol transport in mammalian cells. Cell.

[49] Weiss B, Stoffel W (1997). Human and murine serine-palmitoyl-CoA transferase--cloning, expression and characterization of the key enzyme in sphingolipid synthesis. Eur J Biochem.

[50] Hornemann T, Wei Y, von Eckardstein A (2007). Is the mammalian serine palmitoyltransferase a high-molecular-mass complex?. Biochem J.

[51] Jiang Y (2020). Ceramide subclasses identified as novel lipid biomarker elevated in women with polycystic ovary syndrome: a pilot study employing shotgun lipidomics. Gynecol Endocrinol.

[52] Condorelli RA (2017). PCOS and diabetes mellitus: from insulin resistance to altered beta pancreatic function, a link in evolution. Gynecol Endocrinol.

[53] Mandal N (2021). Role of ceramides in the pathogenesis of diabetes mellitus and its complications. J Diabetes Complications.

[54] Zhou J (2021). miR-206 serves an important role in polycystic ovary syndrome through modulating ovarian granulosa cell proliferation and apoptosis. Exp Ther Med.

[55] Zhang Z (2021). Differential expression of long non-coding RNA regulator of reprogramming and its molecular mechanisms in polycystic ovary syndrome. J Ovarian Res.

[56] Jiang B (2020). Upregulation of microRNA-204 improves insulin resistance of polycystic ovarian syndrome via inhibition of HMGB1 and the inactivation of the TLR4/NF-κB pathway. Cell Cycle.

[57] Bas D (2011). Altered expression of Bcl-2 and Bax in follicles within dehydroepiandrosterone-induced polycystic ovaries in rats. Cell Biol Int.

[58] Maxel T (2017). Expression patterns and correlations with metabolic markers of zinc transporters ZIP14 and ZNT1 in obesity and polycystic ovary syndrome. Front Endocrinol.

[59] Dunaif A (1995). Excessive insulin receptor serine phosphorylation in cultured fibroblasts and in skeletal muscle. A potential mechanism for insulin resistance in the polycystic ovary syndrome. J Clin Invest.

[60] Diamanti-Kandarakis E, Dunaif A (2012). Insulin resistance and the polycystic ovary syndrome revisited: an update on mechanisms and implications. Endocr Rev.

[61] Wehr E (2011). Vitamin D-associated polymorphisms are related to insulin resistance and vitamin D deficiency in polycystic ovary syndrome. Eur J Endocrinol.

[62] Lässer C, Eldh M, Lötvall J (2012). Isolation and characterization of RNA-containing exosomes. J Vis Exp.

[63] Purushothaman A (2019). Exosomes from cell culture-conditioned medium: isolation by ultracentrifugation and characterization. Methods Mol Biol.

[64] Chang X (2018). Exosomes from women with preeclampsia induced vascular dysfunction by delivering sFlt (Soluble Fms-Like Tyrosine Kinase)-1 and sEng (Soluble Endoglin) to endothelial cells. Hypertension.

[65] Jia L (2018). Maternal and umbilical cord serum-derived exosomes enhance endothelial cell proliferation and migration. FASEB J.

[66] Langmead B (2009). Ultrafast and memory-efficient alignment of short DNA sequences to the human genome. Genome Biol.

[67] Ross PL (2004). Multiplexed protein quantitation in Saccharomyces cerevisiae using amine-reactive isobaric tagging reagents. Mol Cell Proteomics.

[68] Wang LH (2007). pFind 2.0: a software package for peptide and protein identification via tandem mass spectrometry. Rapid Commun Mass Spectrom.

[69] Anders S, Huber W (2010). Differential expression analysis for sequence count data. Genome Biol.

[70] Chi H (2015). pFind-Alioth: a novel unrestricted database search algorithm to improve the interpretation of high-resolution MS/MS data. J Proteomics.

